# SH003 induces apoptosis of DU145 prostate cancer cells by inhibiting ERK-involved pathway

**DOI:** 10.1186/s12906-016-1490-5

**Published:** 2016-12-07

**Authors:** Yu-Jeong Choi, Youn Kyung Choi, Kang Min Lee, Sung-Gook Cho, Soo-Yeon Kang, Seong-Gyu Ko

**Affiliations:** 1 Department of Cancer Preventive Material Development, Graduate School, Kyung Hee University, Seoul, 02447 Korea; 2 Jeju International Marine Science center for Research & Education, Korea Institute of Ocean Science & Technology (KIOST), Jeju, 63349 Korea; 3 Department of Science in Korean Medicine, Graduate School, Kyung Hee University, Seoul, 02447 Korea; 4 Department of Biotechnology, Korea National University of Transportation, Jeungpyeong, Chungbuk 368-701 Korea; 5 Department of Preventive Medicine, College of Korean Medicine, Kyung Hee University, 1 Hoegi, Seoul, 130-701 Korea

**Keywords:** SH003, Herbal medicine, Apoptosis, ERK pathway, DU145 human prostate cancer cells, Anticancer effect

## Abstract

**Background:**

Herbal medicines have been used in cancer treatment, with many exhibiting favorable side effect and toxicity profiles compared with conventional chemotherapeutic agents. SH003 is a novel extract from *Astragalus membranaceus*, *Angelica gigas*, and *Trichosanthes Kirilowii Maximowicz* combined at a 1:1:1 ratio that impairs the growth of breast cancer cells. This study investigates anti-cancer effects of SH003 in prostate cancer cells.

**Methods:**

SH003 extract in 30% ethanol was used to treat the prostate cancer cell lines DU145, LNCaP, and PC-3. Cell viability was determined by MTT and BrdU incorporation assays. Next, apoptotic cell death was determined by Annexin V and 7-AAD double staining methods. Western blotting was conducted to measure protein expression levels of components of cell death and signaling pathways. Intracellular reactive oxygen species (ROS) levels were measured using H_2_DCF-DA. Plasmid-mediated ERK2 overexpression in DU145 cells was used to examine the effect of rescuing ERK2 function. Results were analyzed using the Student’s *t*-test and *P*-values < 0.05 were considered to indicate statistically-significant differences.

**Results:**

Our data demonstrate that SH003 induced apoptosis in DU145 prostate cancer cells by inhibiting ERK signaling. SH003 induced apoptosis of prostate cancer cells in dose-dependent manner, which was independent of androgen dependency. SH003 also increased intracellular ROS levels but this is not associated with its pro-apoptotic effects. SH003 inhibited phosphorylation of Ras/Raf1/MEK/ERK/p90RSK in androgen-independent DU145 cells, but not androgen-dependent LNCaP and PC-3 cells. Moreover, ERK2 overexpression rescued SH003-induced apoptosis in DU145 cells.

**Conclusions:**

SH003 induces apoptotic cell death of DU145 prostate cancer cells by inhibiting ERK2-mediated signaling.

**Electronic supplementary material:**

The online version of this article (doi:10.1186/s12906-016-1490-5) contains supplementary material, which is available to authorized users.

## Background

Prostate cancer is the second-most common cancer occurring in men in the United States [1]. Furthermore, the American Cancer Society notes that although the mortality of prostate cancer patients is low, prostate cancer still accounts for a high rate of cancer occurrence [2]. Therefore, the development of new drugs should aid prostate cancer patients.

Almost all prostate cancers develop from glandular cells, and are therefore classified as adenocarcinomas. Prostate cancer cells are also classified into androgen-dependent and-independent groups. While apoptosis of androgen-dependent cells is induced by androgen ablation, other mechanisms are required to induce apoptosis of androgen-independent cells [3, 4]. Furthermore, androgen-independent cells proliferate rapidly and their presence is associated with a poor prognosis [5, 6]. Therefore, the development of novel therapeutic strategies to treat androgen-unresponsive prostate cancer cells is required.

Commonly used prostate cancer cell lines include DU145, PC-3, and LNCaP cells, which have been derived from metastatic prostate cancer lesions from brain, bone, and lymph node tissues, respectively [7]. Because these cell lines originate from different organs and their associated tumor microenvironments are disparate, each cell line has distinct characteristics [8]. DU145 cells are androgen-insensitive and express prostate-specific antigen. These characteristics are typical of difficult-to-treat prostate cancer lesions.

The mitogen-activated protein kinases (MAPKs) are a family of kinases that modulate cell proliferation, survival, differentiation, and development [9, 10]. MAPK pathways transduce signals from extracellular stimuli such as growth factors and mitogens to the nucleus [11–13]. Aberrant signaling of the MAPK ERK is tightly associated with cancer progression [14, 15]. Moreover, overexpression of ERK predicts a poor prognosis in many cancers [16, 17]. Thus, ERK-mediated signaling represents a molecular target for cancer treatment [18]. Furthermore, many natural products exert anti-cancer effects through inhibiting ERK-mediated signaling [7, 8, 19].

Many patients have a growing interest in phytomedicines. Conventional chemotherapeutic agents can have significant side effects, and interest in the use of natural herbal medicines in the prevention and/or treatment of many diseases including cancer has been increasing [20, 21]. SH003 is an extract from *Astragalus membranaceus* (Am), *Angelica gigas* (Ag), and *Trichosanthes Kirilowii Maximowicz* (Tk). The herbal components of SH003 exhibit anti-cancer effects [19, 22, 23], and suppress breast cancer growth [24]. In the present study, we investigated whether SH003 exerts anti-cancer effects on human prostate cancer cells. We report that SH003 induces apoptotic cell death in DU145 prostate cancer cells through inhibiting ERK-mediated signaling.

## Methods

### Preparation of SH003

SH003 was extracted from Am (333 g), Ag (333 g), and Tk (333 g) at a 1:1:1 ratio, according to the principles of traditional Korean medicine. Each component underwent sensory evaluation by Korean Pharmacopoeia standards. Am and Tk were from China, and Ag was of Korean origin. These extracts were concentrated under reduced pressure at ≤ 60 °C and were obtained from Hanpoong Pharm and Foods Company (Jeonju, Korea) [10, 24]. Dry powders were dissolved in 30% ethanol and were prepared as final stock concentrations of 20 mg/mL.

### Cell culture and viability assay

DU145 human prostate cancer cells were cultured in RPMI-1640 medium containing 10% fetal bovine serum and 1% antibiotic. Cells were maintained in a humidified atmosphere with 5% CO_2_ at 37 °C. Cell viability was measured using the MTT assay (Sigma-Aldrich, USA). Cells were seeded on 96-well plates and treated with various concentrations of herbal extract for 72 h. After treatment, MTT working solution was added and cells were incubated at 37 °C for a further 2 h. Next, dimethyl sulfoxide was added to each well to dissolve the formazan crystals. The absorbance of each well was measured at 570 nm using an ELISA reader (Molecular Devices, Palo Alto, CA).

### Apoptosis analysis by flow cytometry

Apoptotic cell death was determined by flow cytometry following Annexin V/7-AAD double staining. Cells were seeded and treated with various concentrations of SH003 for 48 h. After treatment, cells were harvested, resuspended in binding buffer, and stained with Annexin V and 7-AAD. Flow cytometry was conducted using a FACSCalibur instrument (BD Biosciences, San Jose, CA, USA). Data were analyzed using CellQuest Pro software (BD Biosciences).

### Cell proliferation assay

Cell proliferation was measured by labeling cells with bromodeoxyuridine (BrdU) and propidium iodide (PI) prior to flow cytometry. BrdU-positive cells and PI staining were used to identify cells in S phase and expression of total DNA [25, 26]. Cells were treated with SH003 for 48 h and labeled with 10 μM BrdU (Sigma-Aldrich) for 1 h before harvesting. Cells were then trypsinized and fixed in 70% ethanol on ice for 20 min. Next, cells were incubated with 2 M HCl/0.5% Tween-20/phosphate-buffered saline (PBS) for 30 min at room temperature. After washing with 1% bovine serum albumin (BSA) in PBS, cells were stained with anti-BrdU antibody (1:50; Santa Cruz, CA, USA) in buffer (0.5% Tween-20/1% BSA in PBS) for 30 min at room temperature. Cells were washed and then incubated for 30 min at room temperature with goat anti-mouse IgG-FITC (1:100; Santa Cruz). Washed cells were resuspended in PI for 30 min on ice. Cell proliferation was analyzed by FACSCalibur using CellQuest Pro software.

### Western blot analysis

DU145 cells were lysed in radioimmunoprecipitation assay buffer (150 mM NaCl, 1% Triton X-100, 1% sodium deoxycholate, 0.1% sodium dodecyl sulfate, 50 mM Tris–HCl [pH 7.5], 2 mM ethylenediaminetetraacetic acid) and 15 μg of protein was separated on 6–12% gels by sodium dodecyl sulfate-polyacrylamide gel electrophoresis Proteins were transferred to polyvinylidene difluoride membranes and then membranes were blocked in PBS with 0.1% Tween-20 containing 1% BSA and 1.5% skim milk for 1 h. After washing, the membranes were probed with primary antibody at 4 °C overnight, and then incubated with horseradish peroxidase-conjugated secondary antibody for 1 h at room temperature. The blot was developed using the EZ-western detection kit (Daeillab Service, Co., Seoul, Korea). Anti-cleaved caspase-8, −cleaved caspase-3, −PARP, −JNK, −p38, −p-ERK1/2, −p-SRC (Tyr-416 and Tyr-527), −SRC, −p-STAT3, −STAT3, −p-PI3K, −PI3K, −p-AKT (Ser-473), −AKT, −Ras, −p-Raf1 (Ser-259 and Ser-338), −p-MEK1/2, −p-p90RSK (Ser-380), and -RSK1/RSK2/RSK3 antibodies were purchased from Cell Signaling Technology (Danvers, MA, USA). Anti-β-Actin, −p-JNK, −p-p38, −ERK2, and -Raf1 antibodies were obtained from Santa Cruz Biotechnology (Santa Cruz). The band intensities of specific antibodies were normalized and analyzed by ImageJ (Broken Symmetry Software, version 1.4.3.67).

### ROS measurement

Intracellular levels of reactive oxygen species (ROS) were measured by flow cytometry. First, cells were seeded and treated with SH003 for 1 h. In parallel with drug treatment for 1 h, cells were stained using 2′-7′-dichlorodihydrofluorescein diacetate (H_2_DCF-DA) fluorescent dye to measure ROS production for 1 h at 37 °C. Cells were harvested, washed with PBS, filtered, and analyzed by FACSCalibur. N-acetyl-L-cysteine (NAC) was used as ROS scavenger.

### Transfections

Cells were seeded and transfected with pEGEP-C1 or pEGEP-C1-ERK2 plasmids using Lipofectamine 2000 (Invitrogen, Carlsbad, CA, USA). Transfected cells were used for western blotting and apoptosis analyses.

### Statistical analysis

Data are presented as the means ± standard deviation (SD). Differences between groups were analyzed using a Student’s *t*-test. *P*-values < 0.05 were considered to indicate significant differences.

## Results

### SH003 induces apoptosis in DU145 prostate cancer cells

**Fig. 1:**
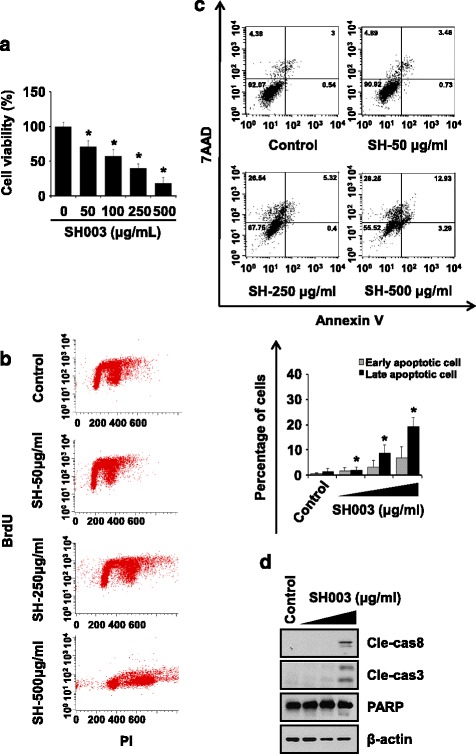
SH003 induces apoptosis in DU145 cells. **a** DU145 cells were exposed to the indicated concentrations of SH003 for 72 h or treated with 30% ethanol as control, and cell viability was measured by the MTT assay. Experiments were performed three times independently and data are presented as the means ± SD.*P < 0.05. **b** Cells were treated with SH003 for 48 h and double-stained with anti-BrdU and propidium iodide (PI). Cell proliferation was determined using cells stained with BrdU for DNA synthesis and DNA content was detected by PI. The dot blots display BrdU incorporation (Y-axis) and DNA content (X-axis). **c** DU145 cells were exposed to SH003 for 48 h and then stained with Annexin V and 7-AAD before flow cytometry. Data are presented as the means ± SD and experiments were replicated three times. *P < 0.05. **d** Levels of apoptosis-related proteins were detected by western blotting with indicated antibodies after treatment with SH003 for 24 h. β-actin was used as a loading control. Black triangle indicates increasing concentrations of SH003 (50, 250, and 500 μg/mL)

We first investigated the effects of SH003 on the viability of DU145 prostate cancer cells using MTT assays. Cells were exposed to 0, 50, 100, 250, or 500 μg/mL SH003 for 72 h. SH003 decreased cell viability in a dose-dependent manner (Fig. 1a). BrdU incorporation assays revealed that SH003 reduced the percentage of BrdU-positive cells (Fig. 1b). These findings indicate that SH003 reduces DU145 cell viability.

We next treated DU145 cells with SH003 for 48 h and found that the number of apoptotic cells (Annexin V^+^/7-AAD^−^ and Annexin V^+^/7-AAD^+^) increased in a dose-dependent manner (Fig. 1c). Consistently, SH003 induced cleavage of caspase-8, caspase-3, and PARP (Fig. 1d). These data indicate that SH003 induced apoptotic cell death in DU145 cells. Likewise, SH003 also induced apoptosis in both LNCaP and PC-3 cells (Additional file 1: Figure S1), suggesting that SH003 can induce apoptosis in multiple prostate cancer cell subtypes.

### Intracellular ROS generation is not involved in SH003-mediated apoptosis

**Fig. 2:**
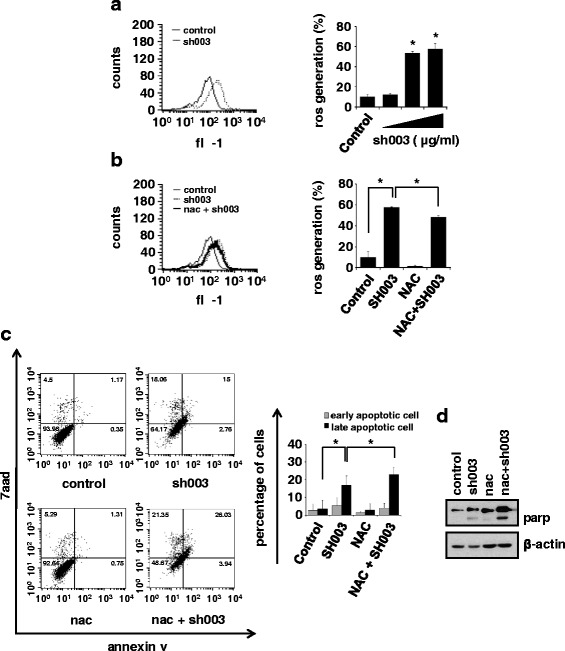
Effects of intracellular ROS generation on apoptotic cell death of DU145. **a** Cells were treated with 50, 250, or 500 μg/mL SH003 and H_2_DCF-DA dye for 1 h at 37 °C. **b** Cell were pretreated with 3 mM NAC for 30 min and then treated with 500 μg/mL of SH003. ROS generation in DU145 cells was measured by flow cytometry. Experiments were repeated three times. *P < 0.05. **c** Cells were pretreated with 3 mM of NAC for 1 h and then treated with 500 μg/mL SH003 for another 48 h. Apoptosis was detected by flow cytometry and data are presented as the means ± SD. Experiments were performed in triplicate. *P < 0.05. **d** Apoptosis-related protein levels were detected by western blotting

ROS induces apoptotic cell death [27–29], so we next examined whether treatment with SH003 increased intracellular ROS levels. SH003 induced ROS production in a dose-dependent manner (Fig. 2a). Moreover, SH003-induced increased intracellular ROS levels decreased when cells were treated with NAC (Fig. 2b). However, combination treatment with SH003 and NAC increased apoptosis (Fig. 2c and d), indicating that SH003-mediated apoptosis did not require intracellular ROS generation.

### SH003-induced JNK phosphorylation is not required for apoptotic cell death

**Fig. 3:**
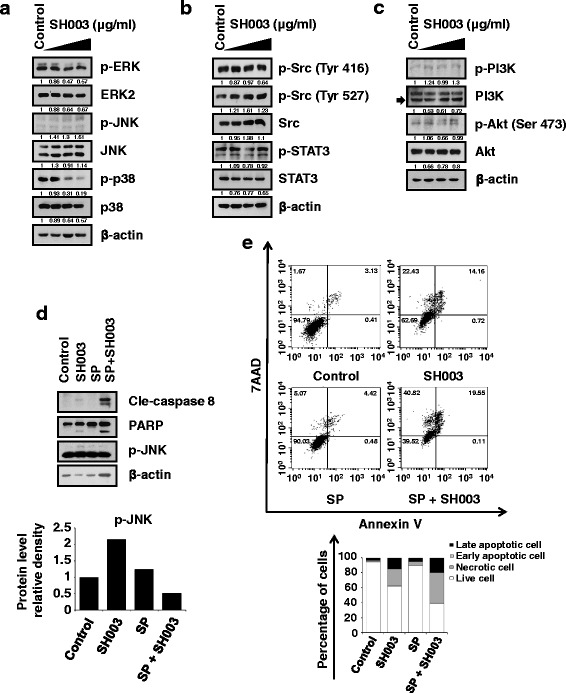
Effects of SH003 on the activation of intracellular signaling pathways and induction of apoptosis by JNK phosphorylation in DU145 cells. DU145 cells were exposed to 50, 250, or 500 μg/mL SH003 for 15 min and western blotting was used to determine expression levels of **a** ERK, JNK, and p38 related MAPK components, **b** SRC-STAT3 signaling pathway components, or **c** PI3K-AKT pathway proteins. β-actin served as the internal control. **d** Cells were pretreated with 10 μM SP600125 for 30 min and then treated with 500 μg/mL SH003 for 24 h. Levels of apoptosis-related proteins and p-JNK were measured by western blotting. **e** Cells were pretreated with 10 μM of SP600125 for 30 min and then treated with 500 μg/mL SH003 for 48 h before staining with Annexin V and 7-AAD at room temperature in the dark. Levels of apoptosis were analyzed by flow cytometry. *P < 0.05

We next examined the intracellular signaling pathways induced by SH003. SH003 reduced phosphorylation of both ERK and p38MAPK, but induced JNK phosphorylation (Fig. 3a). However, SH003 did not affect phosphorylation of SRC, STAT3, PI3K, or AKT (Fig. 3b and c). While our previous work found that SH003 inhibits STAT3 in MDA-MB-231 breast cancer cells [24], this did not occur in DU145 prostate cancer cells. Therefore, SH003 may inhibit the growth of different cancer cells using cell-specific mechanisms.

Because SH003 activated JNK phosphorylation in DU145 cells, we next investigated whether SH003-mediated apoptosis is regulated by JNK signaling. When DU145 cells were treated with the JNK1/2 inhibitor SP600125 prior to SH003 treatment, apoptotic cell death was increased (Fig. 3d and e). These results indicate that SH003-induced JNK phosphorylation is not associated with SH003-mediated induction of apoptosis in DU145 cells.

### SH003 inhibits ERK phosphorylation to promote apoptotic cell death

**Fig. 4:**
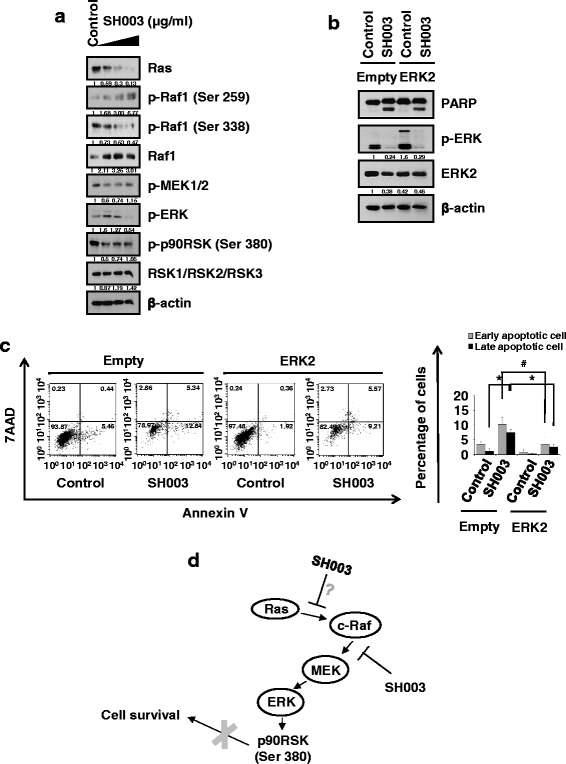
SH003 induces apoptotic cell death in DU145 cells via the ERK signaling pathway. **a** Western blotting of ERK signaling-related protein expression levels in DU145 cells treated with indicated concentrations of SH003. **b** Western blotting of ERK2 and PARP levels in DU145 cells transfected with ERK2-expressing or control vectors and treated with indicated concentration of SH003 for 24 h. **c** Flow cytometry analysis of apoptotic cell death of DU145 cells transfected with the ERK2-expressing or control vectors. Results are presented as the means ± SD of three independent experiments. *P < 0.05 comparison of control vector-transfected cells with ERK2-transfected cells treated with SH003 in late apoptosis, #*P* < 0.05 comparison of ERK2-transfected cell with SH003 treatment in early apoptotic cells. **d** Schematic of the hypothetical system regulated by SH003. SH003 induces apoptosis in prostate cancer cells and inactivates ERK signaling

We next examined whether SH003 regulates the ERK signaling pathway. SH003 reduced phosphorylation of Raf1, MEK, ERK, and p90RSK and decreased Ras levels (Fig. 4a). Furthermore, exogenous expression of ERK in DU145 cells decreased SH003-induced PARP cleavage (Fig. 4b). Accordingly, ERK overexpression reduced SH003-mediated apoptosis based on Annexin V/7-AAD staining (Fig. 4c). These data indicate that SH003 induced apoptotic cell death of DU145 cells by inhibiting ERK-associated signaling (Fig. 4d). However, SH003 did not alter the ERK phosphorylation status in LNCaP and PC-3 cells (Additional file 2: Figure S2). This suggests that SH003 induced apoptosis by intracellular mechanisms other than ERK signaling in these cells.

## Discussion

SH003 is a modified traditional herbal medicine that we have recently developed and used to inhibit breast cancer growth and metastasis [24, 30–32]. Our present findings demonstrate that SH003 can also cause the apoptotic cell death of prostate cancer cells. We found that SH003 induced apoptosis in DU145, LNCaP, and PC-3 prostate cancer cells. These findings suggest that the pro-apoptotic effects of SH003 are not restricted to specific prostate cancer cell types. Taken together with our recent studies describing SH003-mediated breast cancer cell death [24, 30, 33], these results suggest that the anti-cancer effect of SH003 may also not be limited to cancer types.

SH003 increased intracellular ROS levels in DU145 cells. However, while excessive ROS levels can induce apoptosis [28, 34], our data reveal that SH003 induces apoptosis of DU145 cells independently of ROS levels. Although we still do not know the role of SH003-mediated increased ROS levels in DU145 cells, it is possible that the levels of ROS present may be insufficient to directly induce apoptosis. Additionally, SH003-mediated JNK phosphorylation was also not associated with SH003-mediated apoptosis of DU145 cells. Therefore, the precise role of SH003-induced JNK activation remains to be determined.

Our data show that SH003-mediated inhibition of ERK phosphorylation is crucial for apoptotic cell death, with these findings confirmed by rescue experiments. We have also recently shown that SH003 inhibits VEGFR phosphorylation in VEGF-stimulated endothelial cells [32]. Moreover, SH003 also reduces EGFR phosphorylation in MDA-MB-231 breast cancer cells. Presently, we found that SH003 inhibits Raf phosphorylation. Inhibition of EGFR signaling is an effective strategy for prostate cancer treatment [35–37]. While additional findings and supportive data are required to better understand the mechanisms by which SH003 functions, it is possible that SH003 directly regulates receptor tyrosine kinase-mediated signaling in cancer. Our future studies will focus on this issue. Our present findings indicate that SH003-induced ERK inhibition-mediated apoptosis is limited to DU145 cells. These data suggest that SH003 may evoke different intracellular signaling mechanisms to effect its anti-cancer functions.

## Conclusions

SH003 causes apoptosis of DU145 prostate cancer cells by inhibiting ERK signaling. While a more complete understanding of how SH003 differentially affects different subsets of prostate cancer cell populations is still required, this study suggests that SH003 could be beneficial for treating prostate cancer.

## Additional files

Additional file 1: Figure S1. Effects of SH003 on apoptosis in LNCaP and PC-3 cells. **a** LNCaP and PC-3 cells were treated to the indicated concentrations with SH003 for 72 h and with 30% ethanol as control. Cell viability was measured by the MTT assay. Data represents the mean SD.**P,*< 0.05. **b** Cells were exposed to SH003 for 48 h. The harvested-cells were double-stained with Annexin V and 7-AAD for 15 min at RT in the dark. The apoptotic cells were analyzed by FACSCalibur. Data represents the mean ± SD. **c** After treatment with SH003 for 24 h, the apoptosis-related protein levels were confirmed by western blot. β-actin was used as a loading control. Black triangle means concentrations of SH003 (50, 250, 500 μg/ml). (PDF 118 kb)
Additional file 2: Figure S2. Regulation of protein expression of SH003 on ERK signaling pathway in LNCaP and PC-3 cells. Cells were treated with SH003 for 15 min and then detected ERK-related protein expression levels by western blots. Protein expression levels shown above were quantified using ImageJ. (PDF 111 kb)

## Abbreviations

7-AAD: 7-Aminoactinomycin D
Ag: *Angelica gigas*
Am: *Astragalus membranaceus*
H_2_DCF-DA: 2′-7′-dichlorodihydrofluorescein diacetate
MAPK: Mitogen-activated protein kinase
MTT: 3-(4,5-dimethylthiazol-2-yl)-2,5-diphenyltetrazolium bromide
ROS: Reactive oxygen species
SD: Standard deviation
Tk: *Trichosanthes Kirilowii Maximowicz*
