# Supplementary figures and images for: SH003 induces apoptosis of DU145 prostate cancer cells by inhibiting ERK-involved pathway
Source: BMC Complement Altern Med. 2016 Dec 7;16:507. doi: 10.1186/s12906-016-1490-5 (PMC5142381; doi:10.1186/s12906-016-1490-5)

**a**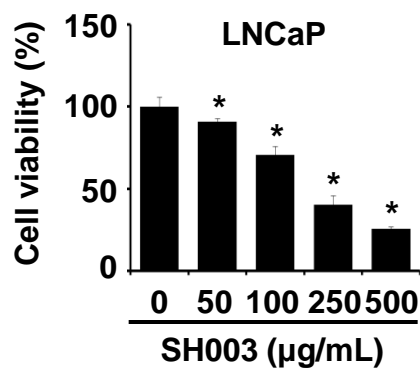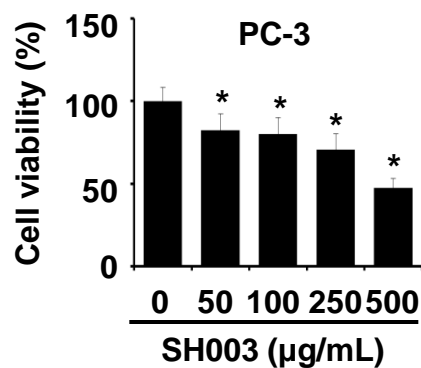**b**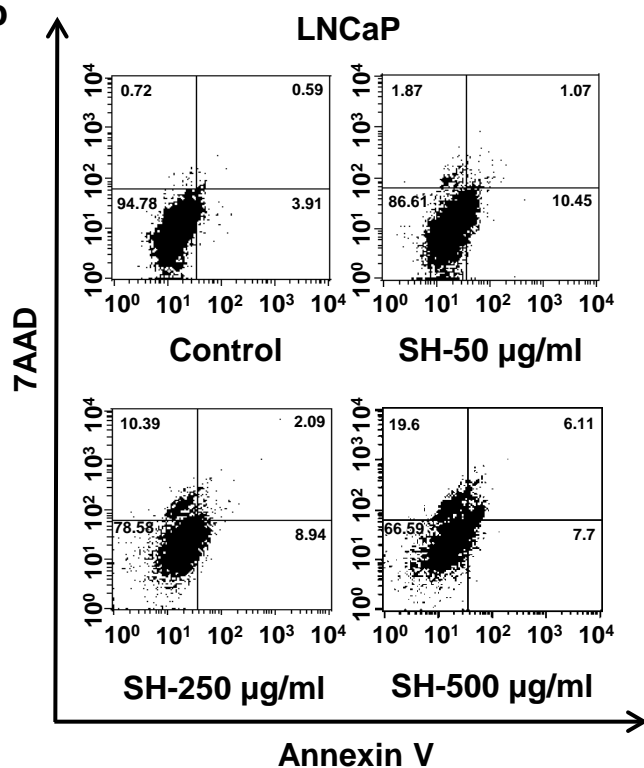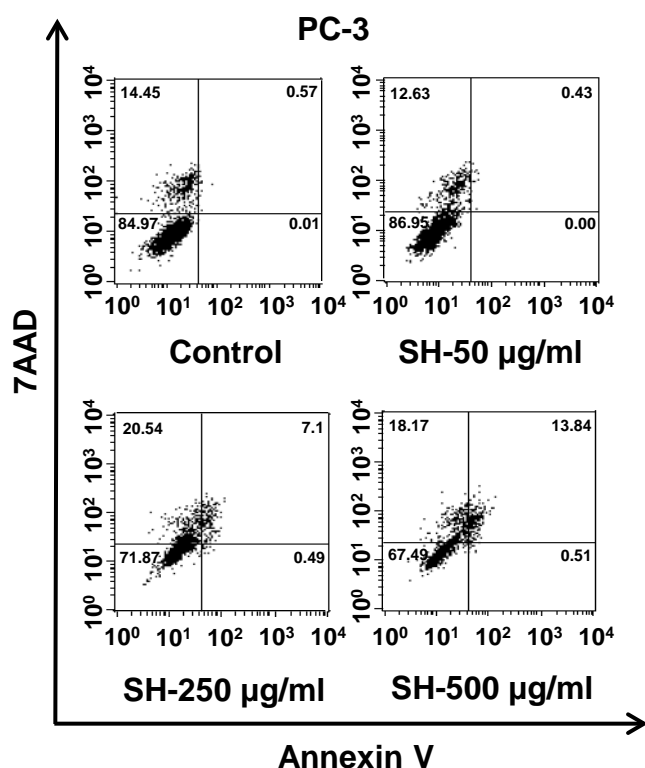**c**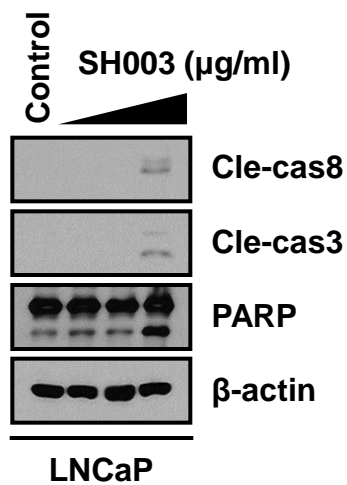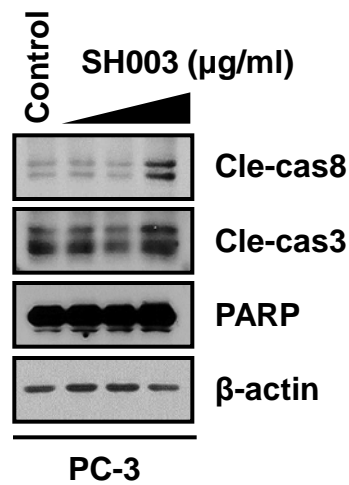

Supplement: Additional file 1: Figure S1. — Effects of SH003 on apoptosis in LNCaP and PC-3 cells. a LNCaP and PC-3 cells were treated to the indicated concentrations with SH003 for 72 h and with 30% ethanol as control. Cell viability was measured by the MTT assay. Data represents the mean SD.*P,< 0.05. b Cells were exposed to SH003 for 48 h. The harvested-cells were double-stained with Annexin V and 7-AAD for 15 min at RT in the dark. The apoptotic cells were analyzed by FACSCalibur. Data represents the mean ± SD. c After treatment with SH003 for 24 h, the apoptosis-related protein levels were confirmed by western blot. β-actin was used as a loading control. Black triangle means concentrations of SH003 (50, 250, 500 μg/ml). (PDF 118 kb) [file 12906_2016_1490_MOESM1_ESM.pdf]

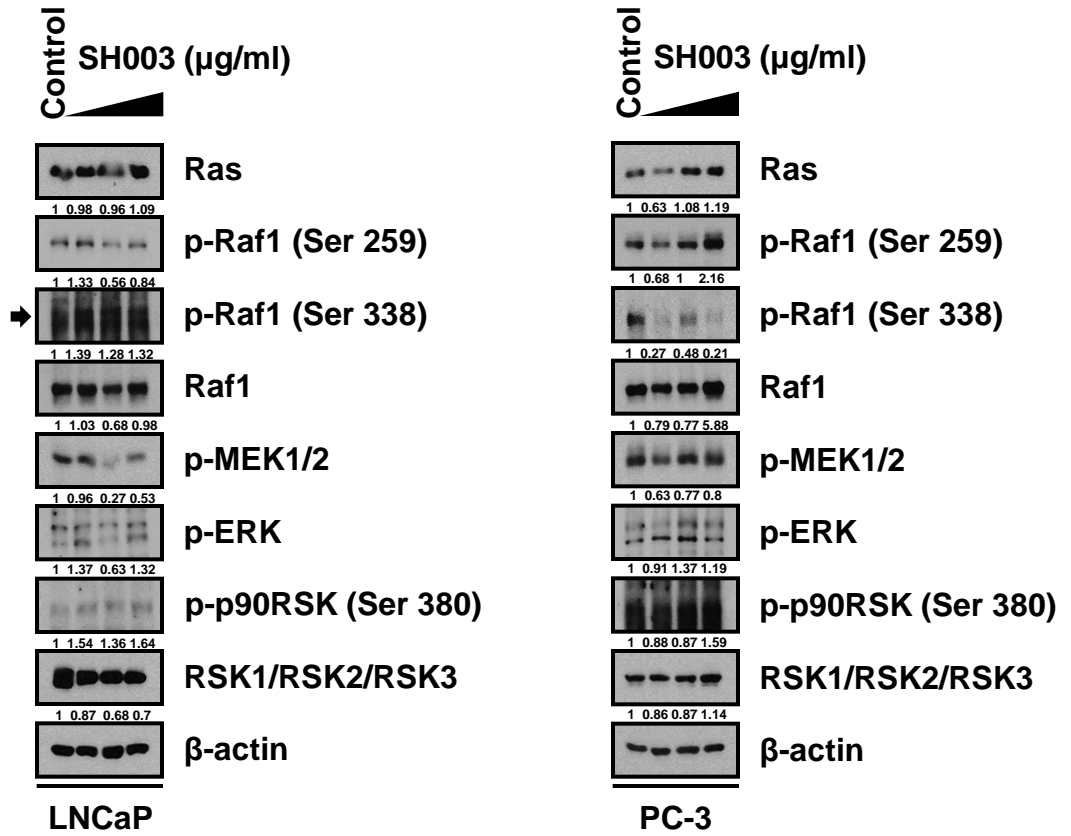

Supplement: Additional file 2: Figure S2. — Regulation of protein expression of SH003 on ERK signaling pathway in LNCaP and PC-3 cells. Cells were treated with SH003 for 15 min and then detected ERK-related protein expression levels by western blots. Protein expression levels shown above were quantified using ImageJ. (PDF 111 kb) [file 12906_2016_1490_MOESM2_ESM.pdf]
